# A nomogram for predicting the risk of cancer-related cognitive impairment in breast cancer patients based on a scientific symptom model

**DOI:** 10.1038/s41598-024-65406-5

**Published:** 2024-06-24

**Authors:** Zhongtao Zhou, Jiajia Ren, Qiankun Liu, Shuoshuo Li, Jiahui Xu, Xiaoyan Wu, Yuanxiang Xiao, Zipu Zhang, Wanchen Jia, Huaiyu Bai, Jing Zhang

**Affiliations:** 1 College of Nursing, Bengbu Medical University, Bengbu, Anhui People’s Republic of China; 2 College of Public Health, Bengbu Medical University, Bengbu, Anhui People’s Republic of China; 3 College of Mental Health, Bengbu Medical University, Bengbu, Anhui People’s Republic of China; 4 College of Clinical Medicine, Bengbu Medical University, Bengbu, Anhui People’s Republic of China

**Keywords:** Breast cancer, Cancer-related cognitive impairment, Symptom Science Model 2.0, Influence factors, Nomogram, Cross-sectional study, Cancer, Psychology

## Abstract

Cancer-related cognitive impairment is a significant clinical challenge observed in patients with breast cancer, manifesting during or after treatment. This impairment leads to deteriorations in memory, processing speed, attention, and executive functioning, which profoundly impact patients' occupational performance, daily living activities, and overall quality of life. Grounded in the Symptom Science Model 2.0, this study investigates the contributing factors to Cancer-related cognitive impairment in breast cancer patients and develops a predictive nomogram for this demographic. Employing both univariate and multivariate logistic regression analyses, this investigation delineates the predictive factors influencing outcomes in breast cancer patients. A nomogram was constructed leveraging these identified predictive factors, accompanied by internal validation through bootstrap resampling methodology (1000 bootstrap samples). The efficacy of the predictive model was assessed by employing the Hosmer–Lemeshow goodness-of-fit test and calibration curves. The prevalence of cognitive impairment in breast cancer patients was identified to be 45.83%.Multivariate logistic regression analysis identified the independent predictors of Cancer-related cognitive impairment in breast cancer patients as place of residence, educational level, chemotherapy, benefit finding, post-traumatic growth, anxiety, fear of cancer progression, and fasting blood glucose levels. these factors were integrated into the nomogram. The Hosmer–Lemeshow goodness-of-fit test demonstrated that the prediction model was appropriately calibrated (χ^2^ = 11.520, *P* = 0.174). Furthermore, the model exhibited an area under the curve of 0.955 (95% CI 0.939 to 0.971) and a sensitivity of 0.906, evidencing its robust discriminative capacity and accuracy. Utilizing the Symptom Science Model 2.0 as a framework, this study comprehensively examines the multifaceted factors influencing Cancer-related cognitive impairment in breast cancer patients, spanning five critical domains: complex symptoms, phenotypic characterization, biobehavioral factors, social determinants of health, and patient-centered experiences. A predictive nomogram model was established, demonstrating satisfactory predictive accuracy and capability. This model is capable of identifying breast cancer patients with cognitive impairments with high precision. The findings furnish empirical evidence in support of the early detection, diagnosis, and intervention strategies for high-risk breast cancer patients afflicted with Cancer-related cognitive impairment.

## Introduction

According to the 2020 Global Cancer Statistics Report, breast cancer has emerged as the most prevalent form of cancer globally, constituting 11.7% of all cancer cases and surpassing lung cancer in incidence^1^. Relative to other malignancies, the prognosis for breast cancer patients is comparatively optimistic. With the continuous advancements in science and technology and the significant improvements in medical standards, treatment plans for breast cancer patients have become increasingly sophisticated, Initially reliant on singular treatment modalities like surgery and radiation therapy, breast cancer management has progressively evolved into the development of personalized plans integrating multiple therapeutic approaches, including chemotherapy^2^, endocrine therapy^3^, targeted therapy^4^, and immunotherapy^5^. the 5-year survival rate for breast cancer patients in the United States has escalated to 91%.In China, the 5-year survival rate for breast cancer patients fluctuates between 71.2 and 74.9%, while in economically advanced cities, it can ascend to as high as 80.9%^6^. Consequently, breast cancer survivors now constitute a significant segment of the cancer survivorship community.

Cancer-related cognitive impairment (CRCI) is increasingly acknowledged as a pivotal symptom experienced by patients both during and after cancer treatment, primarily encompassing cognitive dysfunctions originating from both the malignancy and its therapeutic interventions^7,8^. This condition typically presents as diminished memory, decelerated processing speed, compromised attention, and impaired executive functions. Patients frequently report challenges in thinking, concentrating, and recalling details, which not only precipitate anxiety and depression but also undermine their psychological well-being, profoundly affecting their work capacity, daily living activities, and overall quality of life. Research indicates that 30% to 40% of cancer patients exhibit signs of cancer-related cognitive dysfunction even before undergoing treatment^9^. For patients with non-central nervous system cancers, the incidence of CRCI can ascend to 75% post-treatment, whereas for those with central nervous system cancers, the incidence rate may surge to 90%, underscoring a significant prevalence of cognitive dysfunctions among cancer patients warranting attention^9,10^. Since the 1990s, cognitive dysfunctions in breast cancer patients have been documented, frequently concurrent with postoperative adjuvant chemotherapy. Initial research primarily centered on chemotherapy-related cognitive impairments^11^. However, subsequent research has demonstrated that CRCI extends beyond patients undergoing chemotherapy, indicating that cognitive decline can manifest even before the initiation of chemotherapy. An expanding body of evidence substantiates the presence of CRCI, catalyzing further investigation into this domain^12^. The progression of CRCI is influenced by a multitude of psychosocial and tumor-specific factors. A cross-sectional study conducted by Hajj et al.^13^ involving 112 breast cancer patients revealed that 43.4% experienced depression and 56.2% reported anxiety, indicating a significant correlation between cognitive scores and both anxiety and depression levels. A multicenter longitudinal study has demonstrated that post-traumatic stress disorder (PTSD) induces CRCI^14^. Factors such as anxiety, depression, and PTSD are frequently examined in this area of research, though their predictive roles remain unclear. Furthermore, we found that most current studies on CRCI focus on negative factors or common socioeconomic indices that predict patient outcomes^15^, with a notable lack of further exploration into biomarkers, no studies have conducted a comprehensive and systematic evaluation of breast cancer patients, and the predictors of CRCI complications in these patients from a holistic perspective require further investigation. Therefore, it is of paramount importance to comprehensively evaluate the influencing factors of CRCI in breast cancer patients from multiple perspectives. Understanding these potential factors is crucial for improving the overall prognosis of patients.

In 2015, the National Institute of Nursing Research in the United States first developed Symptom Science Model 1.0 (SSM1.0). As research progressed, by 2019, SSM1.0 had undergone a series of revisions and improvements, evolving into the more refined Symptom Science Model 2.0 (SSM2.0)^16^. This evolution provided a clear conceptual model for symptom science research. This model integrates nursing science and precision health models, advocating for the synergy between biological behavioral factors and symptom science. It integrates potential targeted molecules and biological information with patient symptoms, functional status, and living conditions, providing theoretical support for advancing precise and personalized symptom research to achieve the goal of restoring patient health. SSM2.0 integrates four foundational elements: complex symptoms, phenotypic characterization, biobehavioral factors, and clinical application. Complex symptoms denote a constellation of manifestations impacting the physical, psychological, and social well-being of individuals following acute or chronic disease and injury. Phenotypic characterization encompasses observable traits and features, determined by a combination of behavioral, biological, and clinical data, such as patient behavior characteristics and functional status. Biobehavioral factors encompass the integration of biology, genomics, omics, physiology, and additional clinical laboratory data to identify and refine precise biomarkers. Clinical application propels the translation of research findings into patient care, incorporating evidence-based nursing practices, translational nursing science, intervention strategies, and the evaluation of treatment outcomes. Furthermore, the model incorporates three significant influencing factors: patient-centered experiences, social determinants of health, and policy and population health dynamics. Patient-centered experiences entail the provision of care that respects and caters to the unique preferences, needs, and values of patients in their interactions with the healthcare ecosystem, predominantly involving support from hospitals, communities, families, and groups. Social determinants of health encompass the conditions under which individuals are born, grow, live, work, and age, including the broader socioeconomic, and cultural environment, and background. Policy and population health dynamics scrutinize patient symptoms on a macro scale, employing comprehensive cross-sectional and longitudinal research designs of population health data to elucidate symptom correlations and causations^17^. SSM2.0 has been extensively utilized for symptoms associated with mental illness^18^, highlighting its superiority in symptom-related scientific research. Therefore, Our research integrates five fundamental conceptual elements—complex symptoms, phenotypic traits, biobehavioral factors, patient-centered experiences, and social determinants of health—within the core framework of SSM2.0. Based on these conceptual elements and a comprehensive literature review, a dynamic assessment of breast cancer CRCI was undertaken to thoroughly and precisely identify the predictive factors of CRCI.

The progression of CRCI is influenced by a multitude of psychosocial and tumor-specific factors. Predictive models serve as vital tools for identifying the risk of CRCI in breast cancer, thereby facilitating the implementation of more effective support strategies. Existing predictive models have been effectively applied to various aspects of mental health disorders, including depression and suicide risk^19^. Additionally, a nomogram can simplify traditional predictive model formulas into a single numerical estimate of the probability of an event^20^, thereby aiding clinical decision-making during patient consultations. Currently, some studies aim to predict the cognitive function status of cancer patients; However, these investigations failed to integrate biomarkers to enhance cognitive impairment prognostication. In light of this, the primary objectives of our study are to analyze and identify the variables affecting CRCI in breast cancer using SSM2.0, develop and evaluate predictive models for CRCI in breast cancer, and construct a simplified and efficient nomogram to predict the risk of CRCI occurrence.

## Methods

### Study design and participants

A cross-sectional survey was conducted from June 25, 2023, to January 31, 2024, across seven tertiary hospitals located in the northern Anhui region of China, specifically including institutions from Bengbu, Fuyang, Bozhou, Huaibei, and Suzhou cities. Eligibility criteria for this study required patients to (a) have a diagnosis of breast cancer confirmed through pathological examination; (b) possess no history of other tumors or treatments; (c) have no history of psychiatric medication usage; (d) have no history of organ failure. Exclusion criteria encompassed: the absence of essential pathological and clinical data, history of neurological conditions including Alzheimer's disease, mixed dementia, epilepsy, Parkinson's disease, multiple sclerosis, recent use (within the last month) of sedatives or psychiatric medications, and presence of secondary infections or metastatic tumors. This investigation received approval from the Bengbu Medical University Institutional Review Board (Approval number: 2023–280), with all participants furnishing informed consent.

### Predictive factors

Building upon conceptual elements of the Symptom Science Model (SSM2.0)—complex symptoms, phenotypic characteristics, biobehavioral factors, patient-centered experiences, and social determinants of health—our study conducted an extensive and holistic evaluation of the dynamic symptomatology of CRCI through thorough consultations with neurology experts and an exhaustive review of the literature. The selection of predictive factors was finalized, with 40 pertinent factors being unanimously identified. Further details are as follows: (1) Social determinants of health, encompassing age, marital status, monthly income, years of education, residential area, smoking and drinking habits, physical exercise, smartphone usage, cognitive activities, employment status (inclusive of pre-retirement occupation), surgical history, chemotherapy experience, and comorbidities. (2) Factors pertinent to patient-centered experiences, including social support, benefit finding, post-traumatic growth, and satisfaction with medical care. (3) Complex symptoms incorporate a spectrum of conditions including anxiety, depression, fear of cancer progression, perceived stress, post-traumatic stress disorder (PTSD), psychological distress, and fatigue. (4) Phenotypic characteristics span sleep duration, Body Mass Index (BMI), physical activity levels, hearing status, medication adherence, and menopausal status. (5) Biobehavioral factors encompass biomarkers such as C-reactive protein, hemoglobin levels, blood cholesterol, apolipoproteins, high-density and low-density lipoproteins, total calcium, triglycerides, and fasting blood glucose levels.

### Data collection

A research team was established, comprised of three nursing graduate students and eight undergraduate students. Following the approval of department heads, data collection was executed across multiple hospitals. Data about patients' social determinants of health, biobehavioral factors, and phenotypic characteristics were extracted from the electronic medical record system. Inpatients filled out the questionnaires on-site, whereas outpatients who could not return for follow-up visits were contacted and surveyed via telephone. All data collectors received standardized training and employed uniform instructions to assist patients in completing the questionnaires. After the collection of questionnaires, a dual verification process was conducted by two individuals to eliminate invalid questionnaires, thereby ensuring the authenticity and precision of the research data.

### Research tools

#### Mini-mental State Examination Scale

Originally compiled by Folstein et al. in 1975^21^, this scale has been extensively translated and adapted into various languages, gaining widespread usage^22^. It is recognized as the most widely utilized cognitive function screening scale globally. The scale encompasses five cognitive domains: orientation (10 points), immediate memory (3 points), attention and calculation (5 points), recall (3 points), and language ability (9 points, including naming ability 2 points, repetition ability 1 point, three-step command 3 points, reading ability 1 point, writing ability 1 point, and copying ability 1 point). It comprises a total of 30 items, summing to a maximum score of 30 points. A higher total score signifies superior cognitive function. Based on educational attainment, cognitive impairment is classified as follows: illiterate individuals (0 years of education) are considered unimpaired if scoring above 17; primary school graduates (≤ 6 years of education) if scoring above 20; and those with education beyond junior high school (> 6 years) if scoring above 24. This classification criterion has been empirically validated^23^. The Cronbach's α coefficient of the scale in this study was 0.925.

#### Integrated Data Questionnaire

This questionnaire was developed with guidance from neurology specialists, nursing experts, and statisticians. Drawing upon the SSM2.0 theoretical framework, a selection from the identified 40 predictive factors was made, encompassing variables such as marital status, occupation, smartphone usage, age, education level, individual monthly income, history of chemotherapy, smoking history, treatment regimen, history of alcohol consumption, comorbidities, cognitive activities, satisfaction with medical care, psychological distress (evaluated using a psychological distress thermometer), compliance, language and hearing functions, physical activity capability, sleep quality, BMI, C-reactive protein, hemoglobin, high- and low-density lipoproteins, triglycerides, apolipoprotein B, total cholesterol, calcium levels, and fasting blood glucose.

#### Social Support Rating Scale

Developed by Taiwanese scholar Xiao Shuiyuan^24^, this scale delineates three dimensions: objective support, subjective support, and the utilization of social support, comprising 10 items in total. The aggregate of the scores across all items yields the total social support score, which spans from 12 to 66 points. Higher scores indicate enhanced levels of social support. The Cronbach's α coefficient of the scale in this study was 0.841.

#### Benefit Finding Scale

The Chinese version of the Benefit Finding Scale, adapted by Weaver et al.^25^ and translated by Liu Huichun et al.^26^, was utilized in this study. The scale comprises six dimensions and a total of 22 items, using a Likert 5-level scoring method. Each item is rated from "none" to "very much," corresponding to scores from 1 to 5, respectively. The total score of the questionnaire is the sum of the scores across all dimensions, ranging from 22 to 110 points, with higher scores indicating greater perceived benefits. The Cronbach's α coefficient of the scale in this study was 0.886.

#### Posttraumatic Growth Scale

Developed by Chinese scholar Wang Ji, this scale has been widely applied in research on various trauma patients in China^27^. It comprises five dimensions and contains 20 items: interpersonal relationships, alternative possibilities, personal power enhancement, personal realization, and personal change. Using a Likert 6-level scoring method, each item rang from "no change" to "very much," corresponding to scores from 0 to 5. The total score ranges from 0 to 100 points, with higher scores indicating greater post-traumatic growth. The Cronbach's α coefficient of the scale in this study was 0.782.

#### Fear of Disease Progression Scale

Developed by Mehnert et al. in 2006^28^ and sinicized by Wu Qiyun et al. In 2015^29^, quantifies patients' fear of disease progression. It encompasses two dimensions: physical health, and social and family functions, with a total of 12 items. Using a 5-level Likert scoring method, each item offers five options ranging from "never" to "always," corresponding to scores from 1 to 5. The total score spans from 12 to 60 points, with higher scores signifying a greater fear of disease progression. The Cronbach's α coefficient of the scale in this study was 0.823.

#### Perceived Stress Scale

Developed by Cohen et al. in 1983^25^, This study utilizes the Chinese version of the Perceived Stress Scale, translated by Professor Yang Tingzhong^26^, which is adapted to Chinese cultural and national contexts. The scale comprises 14 items across two dimensions: tension and a sense of loss of control. It employs a 5-level Likert scoring method, with each item offering five options ranging from "never" to "a lot," corresponding to 0 to 4 points. The total score ranges from 0 to 56 points, with higher scores indicating greater perceived stress. The Cronbach's α coefficient of the scale in this study was 0.774.

#### Post-Traumatic Stress Disorder Scale

The scale, developed by the Behavioral Science Division of the PTSD Research Center in the United States according to DSM-IV criteria^27^, was revised by Jiang Chao et al.^28^ for use in Chinese populations. It serves as an effective tool for PTSD screening. The scale consists of 17 items across three dimensions: re-experience, avoidance/numbness, and increased alertness. Each item is scored from 1 to 5, ranging from "not at all" to "very," with a total score ranging from 17 to 85. Higher scores indicate more severe PTSD symptoms. The Cronbach's α coefficient of the scale in this study was 0.932.

#### Hospital Anxiety and Depression Scale

Developed by Zigmond and Snaith in 1983^29^ and revised by Ye Weifei et al. in 1993^30^. It comprises 14 items, with the 7 odd-numbered items forming the anxiety subscale and the 7 even-numbered items forming the depression subscale. Using a 4-point Likert scoring method, each item offers 4 options corresponding to 0 to 3 points. Higher scores indicate more severe symptoms. The Cronbach's α coefficient of the scale in this study was 0.884.

### Statistical analysis

Statistical analyses were meticulously executed utilizing IBM SPSS (version 25.0, https://www.ibm.com/products/spss-statistics) and R software (version 4.3.1; https://cran.r-project.org/bin/windows/base/old/4.3.1/) to ensure rigorous data examination. Univariate analysis was conducted for the predictors, The χ2 test was utilized to analyze categorical variables, the Shapiro–Wilk test to assess the normality of continuous variables, and either the independent *t* test or Mann–Whitney U test to analyze continuous variables. Variables with *P* values ≤ 0.05 in the univariate analysis were included in multivariate logistic regression analysis. Independent risk factors were integrated into the R (version 3.5.2) using the RMS package to construct a predictive nomogram model for cognitive impairment risk in breast cancer patients. Receiver operating characteristic (ROC) curves were plotted, and the area under the curve (AUC) was calculated to evaluate the model’s discriminative efficacy. The model's accuracy was assessed with calibration plots and the Hosmer–Lemeshow goodness-of-fit test. Risk stratification was determined by the optimal cutoff value, calculated at the pinnacle of the Youden index (where Youden index = sensitivity + specificity − 1), representing the probability of BCRL risk. Decision curve analysis was adeptly utilized to appraise the clinical utility of the model, offering insights into practical applications. A two-sided *p* value of less than 0.05 was considered statistically significant, adhering to conventional criteria for hypothesis testing.

### Institutional review board statement

The study was conducted by the Declaration of Helsinki, and approved by the Ethics Committee of Bengbu Medical University (No. 2023-280).

### Informed consent statement

Informed consent was obtained from all subjects involved in the study.

## Result

### Characteristics of participants

Utilizing the SSM2.0 framework, our study constructed Table 1, encompassing comprehensive demographic, clinical, psychosocial, and a multitude of biobehavioral indicators of the participants. These participants were categorized into CRCI and non-CRCI groups based on their cognitive function status. The survey included 515 patients, unveiling a cognitive impairment incidence rate of 45.83%. The CRCI group had an average age of 57 years (interquartile range: 51–66 years), compared to an average age of 52 years (interquartile range: 45–60 years) in the non-CRCI group. Univariate analyses comparing the cognitive impairment cohort with the non-cognitive impairment cohort disclosed significant disparities in health-related social determinants, patient-centered experiences, complex symptoms, phenotypic characteristics, and biobehavioral factors, all substantiated by *p* values < 0.05.

**Table 1:** Clinical characteristics of the study sample (n = 515).

Variables	CRCI		S tatistics	*P*
	NO (n = 279)	Yes (n = 236)		
Age M (P25~P75)	52 (45, 60)	57 (51, 66)	− 5.398 a	< 0.001
Marital status			0.182b	0.670
Married	19 (7)	13 (6)		
Other	260 (93)	223 (94)		
Per capita monthly household income			7.104 b	0.029
≤ 2000 yuan per month	117 (42)	116 (49)		
2000–5000 yuan per month	92 (33)	83 (35)		
≥ 5000元yuan per month	70 (25)	37 (16)		
Education level			80.198 b	< 0.001
0 year	31 (11)	104 (44)		
0–6 years	74 (27)	60 (25)		
> 6 years	174 (62)	72 (31)		
Homeplace			23.651 b	< 0.001
Rural areas	131 (47)	162 (69)		
Urban areas	148 (53)	74 (31)		
Smoking status			0.351 b	0.839
Yes	28 (10)	27 (11)		
Smoking cessation	43 (15)	38 (16)		
No	208 (75)	171 (72)		
Drinking			0.412 b	0.814
No	205 (73)	172 (73)		
Light alcohol drinking	50 (18)	40 (17)		
Heavy alcohol drinking	24 (9)	24 (10)		
Exercise			4.665 b	0.031
Less than 1 time a week	174 (62)	123 (52)		
No	105 (38)	113 (47)		
Smartphone			13.707 b	< 0.001
Yes	239 (86)	170 (72)		
No	40 (14)	66 (28)		
Cognitive activity			12.348 b	< 0.001
Yes	109 (39)	57 (24)		
No	170 (61)	179 (76)		
Occupation (before retirement)			3.070 b	0.08
Low-skilled manual occupation	186 (67)	175 (74)		
Occupations with high responsibilities or knowledge	93 (33)	61 (26)		
Operation			0.946 b	0.331
Yes	173 (62)	157 (67)		
No	106 (38)	79 (33)		
Chemotherapy			4.438 b	0.035
Yes	200 (72)	189 (80)		
No	79 (28)	47 (20)		
Comorbidities			4.174 b	0.041
Yes	86 (31)	94 (40)		
No	193 (69)	142 (60)		
Medical satisfaction			19.465 b	< 0.001
Yes	263 (94)	192 (81)		
No	16 (6)	44 (19)		
Psychological distress			236.480 b	< 0.001
Mild	140 (50)	15 (6)		
Moderate	122 (44)	60 (25)		
Severe	17 (6)	161 (68)		
Fatigue			82.295 b	< 0.001
Yes	70 (25)	154 (65)		
No	209 (75)	82 (35)		
Sleep			52.508 b	< 0.001
≥ 6 h	193 (69)	87 (37)		
< 6 h	86 (31)	149 (63)		
Limb activity			18.497 b	< 0.001
Yes	80 (29)	112 (47)		
No	199 (71)	124 (53)		
Hearing function			10.951 b	< 0.001
Yes	259 (93)	196 (83)		
No	20 (7)	40 (17)		
Compliance			26.220 b	< 0.001
Yes	258 (92)	179 (76)		
No	21 (8)	57 (24)		
Menopause			8.362 b	0.004
Yes	157 (56)	163 (69)		
No	122 (44)	73 (31)		
BMI M (P25~P75)	25.11 (22.48,28.23)	26.23 (23.13,29.2)	− 2.163 a	0.031
Anxiety M (P25~P75)	6 (4, 8)	11 (8, 13)	− 11.120 a	< 0.001
Depression M (P25~P75)	7 (5, 9)	11 (9, 13)	− 11.609 a	< 0.001
Fear of cancer progression M (P25~P75)	30 (24, 36)	38 (31, 44)	− 8.414 a	< 0.001
Perceived stress M (P25~P75)	37 (32.5, 40)	40 (37, 43)	− 6.718 a	< 0.001
Post-traumatic stress disorder M (P25~P75)	29 (23, 35)	47 (34, 54)	− 11.534 a	< 0.001
Social support M (P25~P75)	37 (32, 41)	31 (28, 34)	− 10.774 a	< 0.001
Benefit finding M (P25~P75)	59 (49.5, 68)	42 (38, 50.25)	− 12.125 a	< 0.001
Post-traumatic growth M (P25~P75)	59 (51.5, 64)	34 (17.5, 49)	− 13.733 a	< 0.001
C-reactive protein M (P25~P75)	121 (111, 127)	126 (116, 133)	− 0.849 a	0.396
Hemoglobin M (P25~P75)	121 (111, 127)	126 (116, 133)	− 3.571 a	< 0.001
Blood cholesterol M (P25~P75)	5.09 (4.33, 5.65)	5.12 (4.22, 5.9)	− 0.943 a	0.346
Apolipoprotein M (P25~P75)	0.84 (0.72, 0.98)	0.94 (0.79, 1.06)	− 5.750 a	< 0.001
High-density lipoprotein M (P25~P75)	1.44 (1.22, 1.69)	1.38 (1.22, 1.65)	− 0.991 a	0.322
Low-density lipoprotein M (P25~P75)	2.67 (2.09, 3.21)	3.11 (2.17, 3.39)	− 4.091 a	< 0.001
Total calcium M (P25~P75)	2.3 (2.24, 2.41)	2.28 (2.24, 2.4)	− 1.034 a	0.301
Triglyceride M (P25~P75)	1.59 (1.07, 2.15)	1.8 (1.32, 2.61)	− 3.206 a	0.001
Fasting blood glucose M (P25~P75)	5.2 (4.95, 5.59)	5.48 (4.98, 6.81)	− 3.096 a	0.002

a: Mann–Whitney U test.

b: X2 test.

Furthermore, A comprehensive multicollinearity diagnosis was performed on the continuous independent variables (such as age and social support) intended for the CRCI regression model using SPSS (version 23.0). The results indicated that the variance inflation factors (VIF) for all variables were below 5, suggesting the absence of collinearity among the independent variables. Therefore, it can be concluded that all variables included in the regression analysis are independent.

### Logistic regression analysis of influence factors for CRCI

Based on the univariate analyses, with the occurrence of CRCI as the dependent variable, 32 factors with *P* values less than 0.05 were selected as independent variables for a multivariate logistic regression model. Table 2 presents the logistic regression outcomes, identifying eight significant predictors of CRCI: residential area, education level, benefit finding, post-traumatic growth, chemotherapy, fear of cancer progression, anxiety, and fasting blood glucose levels. These predictors play a crucial role in predicting CRCI outcomes.

**Table 2:** Multivariate logistic regression analysis of predictors for CRCI.

Predictors	Beta coeff	SE	Odds ratio	95% CI*	*P*
(Constant)	2.581	1.382	–	–	0.062
Homeplace (Reference group: rural areas)	− 1.463	0.354	0.232	0.113–0.455	0.000
Education level (Reference group: rural areas)					
0–6 year	− 1.098	0.421	0.333	0.144–0.755	0.009
> 6 years	− (2.270)	0.396	0.103	0.046–0.219	0.000
Chemotherapy (Reference group: No)					
Yes	1.218	0.370	3.380	1.661–7.125	0.001
Benefit finding	− 0.062	0.015	0.939	0.912–0.966	0.000
Post-traumatic growth	− 0.102	0.013	0.903	0.878–0.926	0.000
Anxiety	0.260	0.048	1.298	1.185–1.430	0.000
Fear of cancer progression	0.041	0.021	1.042	1.001–1.086	0.048
Fasting blood glucose	0.507	0.173	1.660	1.190–2.348	0.003

### Development of the prediction model for CRCI

**Figure 1:**
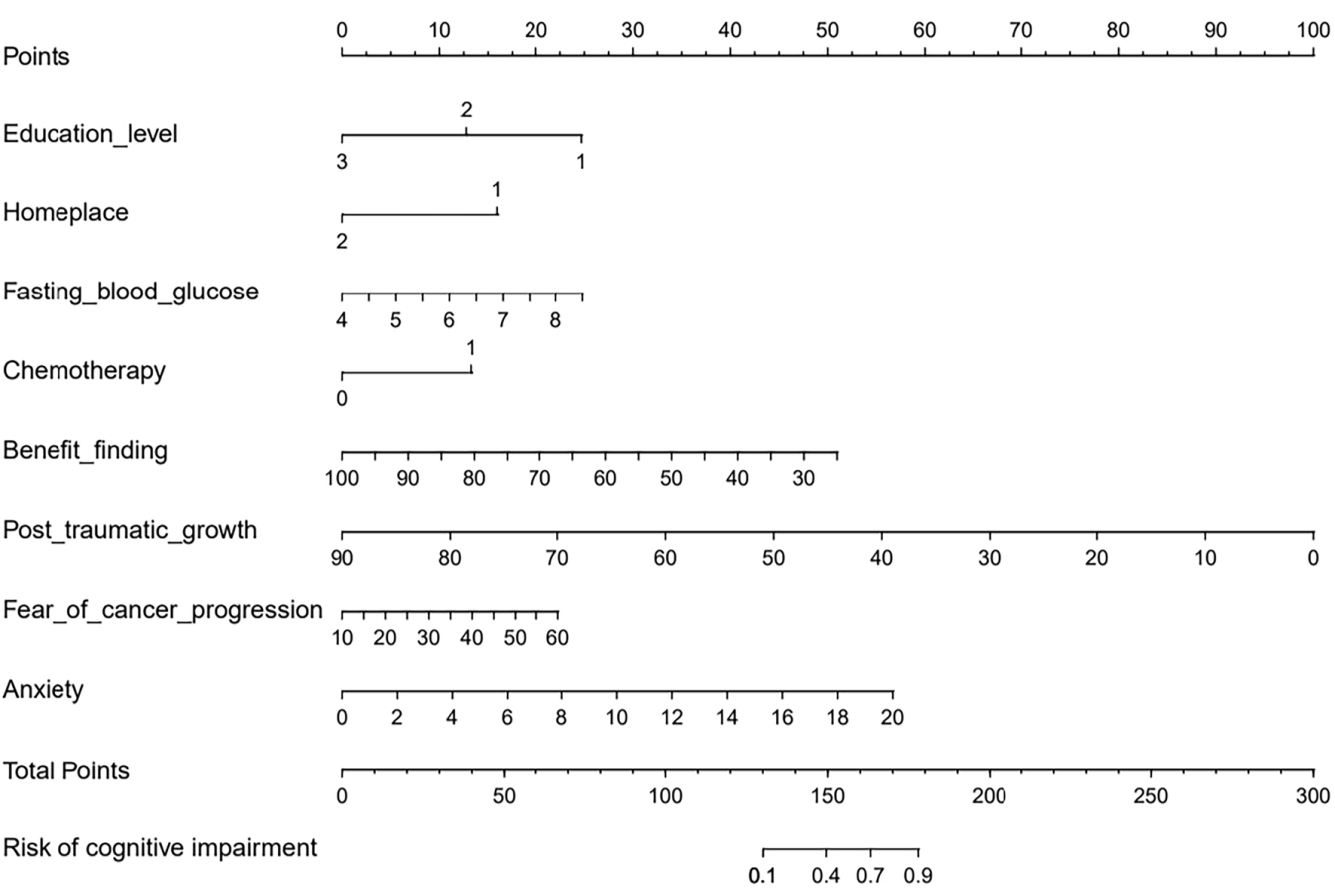
Nomogram to predict the probability of breast cancer-related cognitive disorder in patients with breast cancer.

The eight variables identified as independent risk factors for CRCI were integrated as predictors into the final model, culminating in the development of an individualized nomogram via R software (version 3.5.2), as shown in Fig. 1. The influence of each predictor in the model is quantified based on the regression coefficients derived from the final model, providing a nuanced understanding of their impact. The model elucidates that factors such as residential area, education level, benefit finding, and post-traumatic growth function as protective elements against CRCI among breast cancer patients. Conversely, chemotherapy, fear of cancer progression, anxiety, and fasting blood glucose levels are delineated as risk factors, highlighting the dual nature of influences on patient outcomes.

**Figure 2:**
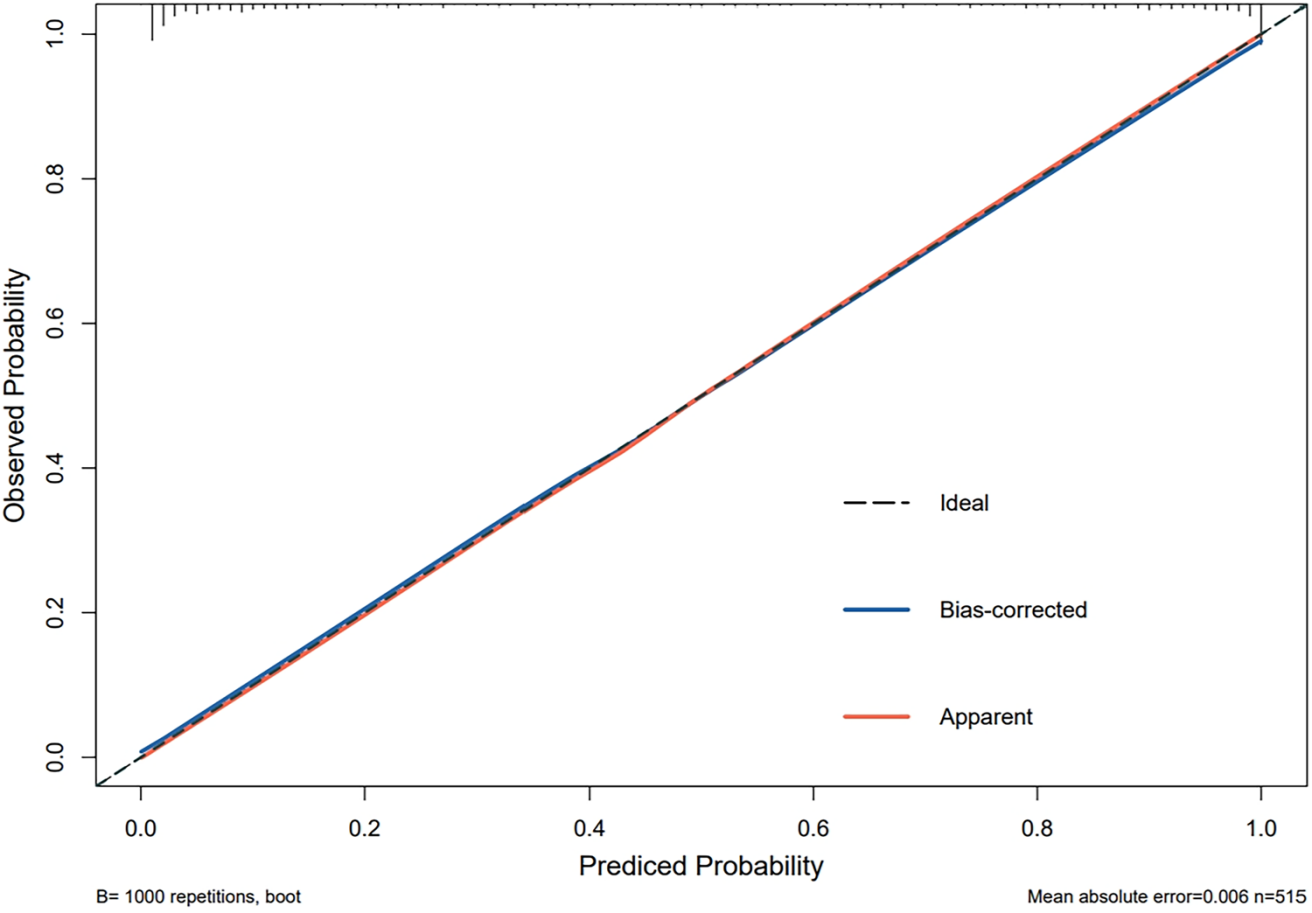
Calibration of the nomogram model for predicting CRCI risk.

**Figure 3:**
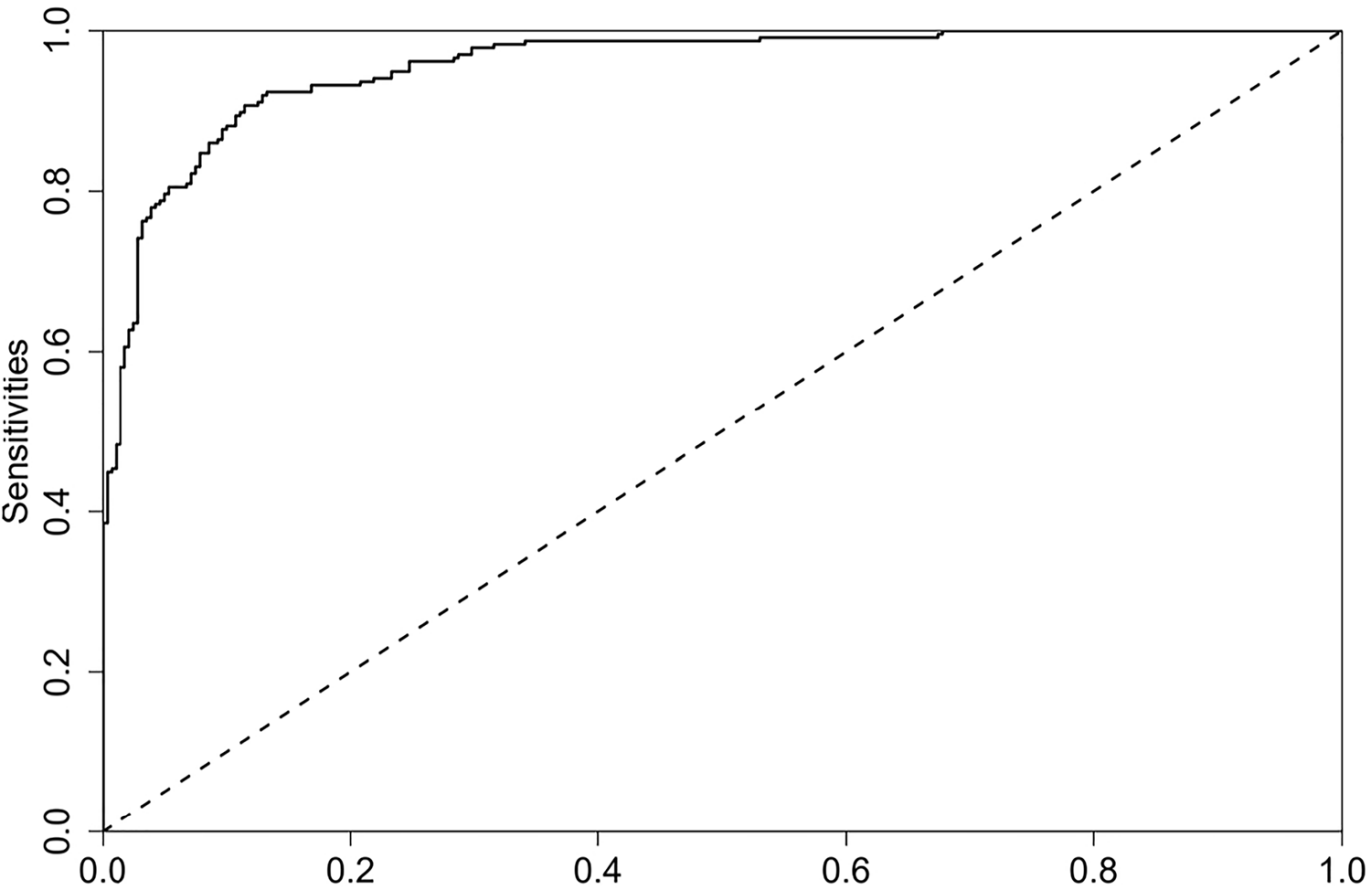
ROC curves for the nomogram model to predict CRCI risk.

**Figure 4:**
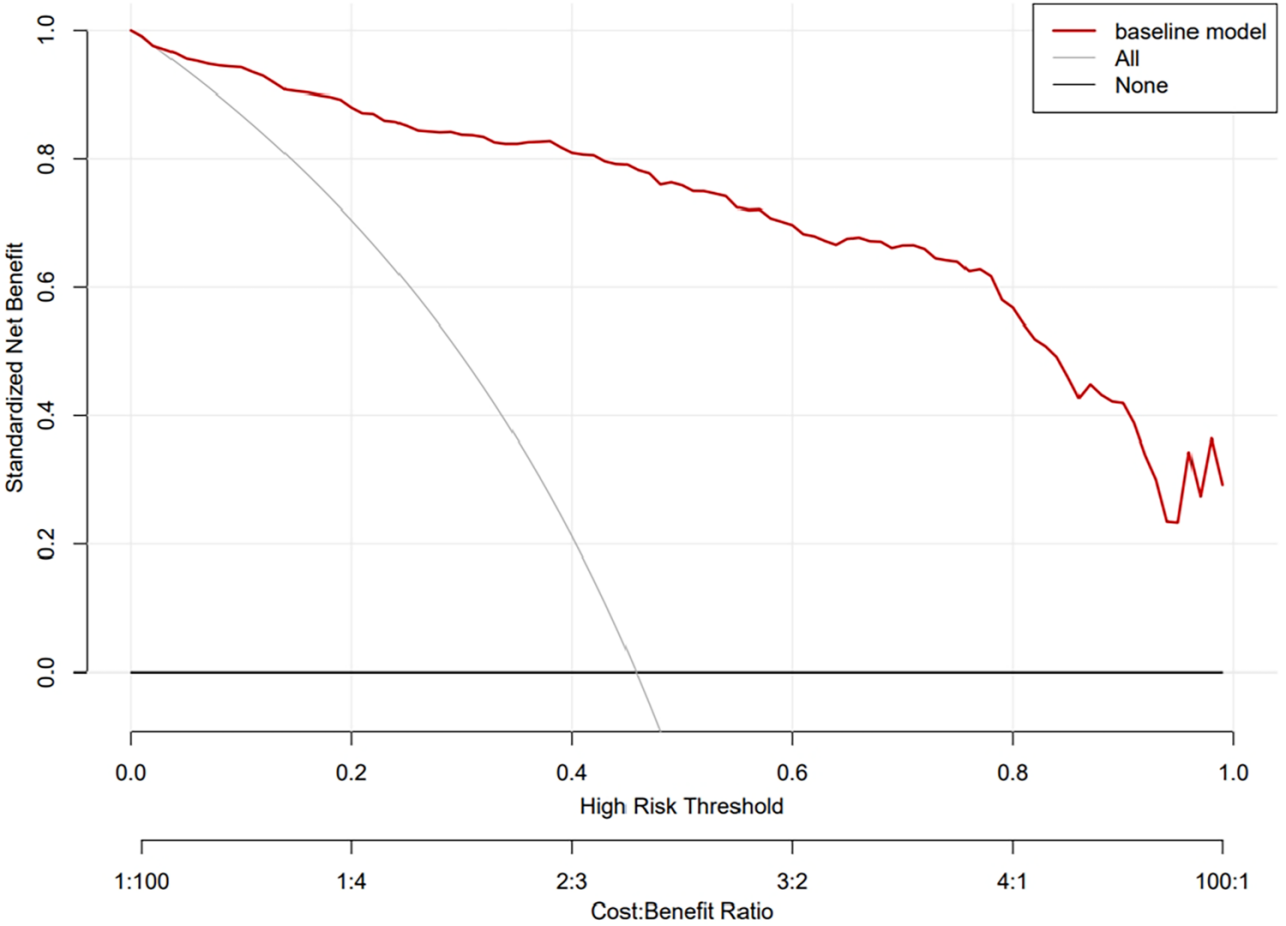
Decision analysis curves for the nomogram model for predicting CRCI risk.

The predictive model for CRCI in breast cancer patients was calibrated using the Hosmer–Lemeshow goodness-of-fit test to ensure accuracy. The test results indicated favorable calibration, with a χ^2^ value of 11.520 and a *P* value of 0.174. The calibration curve (Fig. 2) shows a high degree of concordance between predicted and actual probabilities. Additionally, the ROC curve (Fig. 3) highlights the model's strong discriminative ability, with an AUC value of 0.955 and a 95% confidence interval of 0.939 to 0.971. The decision curve analysis (DCA) (Fig. 4) demonstrates that the model's predictive advantage predominantly surpasses other curves, indicating substantial benefits to patients. The internal validation of the nomogram's predictive accuracy, conducted via the Bootstrap resampling method with 1000 bootstrap resamples, yielded an average AUC value of 0.873.

## Discussion

### Influencing factors of CRCI among Breast *cancer* patients

#### Social determinants of health: homeplace, education level and chemotherapy

In the context of the social determinants of health, residential locality, and educational attainment are crucial factors impacting cognitive impairment in breast cancer patients. Individuals residing in rural areas for prolonged periods exhibit a higher susceptibility to CRCI compared to their urban counterparts. Conversely, patients with higher levels of education have a lower risk of CRCI than those with less education. Residential locality and educational attainment serve as markers of socioeconomic status, with empirical research from the United States and Eastern Europe showing a strong and direct association between these socioeconomic determinants and cognitive function status^31^. This phenomenon can be attributed to the paucity of economic resources and prevalent poverty in specific rural communities, which precipitates a decline in the utilization of medical services and diminished social engagement. This sequence of events hinders the creation of an environment conducive to cognitive stimulation. Consequently, this could lead to an elevated prevalence of cognitive impairment among inhabitants of impoverished rural locales^32^. Educational attainment is regarded as a significant indicator of cognitive reserve. Elevated levels of educational achievement can enhance an individual's cognitive reserve, namely, the brain's ability to process information and counteract cognitive impairment, thereby furnishing early cognitive stimulation and bolstering coping mechanisms. Concurrently, individuals with higher educational backgrounds frequently exhibit enhanced psychological resilience and superior information acquisition and processing capabilities. This empowers them to thoroughly investigate disease and treatment specifics, engage actively in decision-making regarding treatment, sustain elevated levels of health consciousness, and cultivate healthier living habits, consequently facilitating the amelioration of their cognitive functions^33^. The protracted treatment period for breast cancer substantially amplifies the economic strain on average families, thereby intensifying the economic disparities experienced by residents in rural locales and patients with lower educational achievements. Consequently, in the development of health policies intended to bolster cognitive health within the populace, it is imperative to devise targeted and nuanced policy strategies for disparate socioeconomic demographics, paying specific heed to the disparities in urban–rural distribution and educational attainment among breast cancer patients.

Chemotherapy, exhibiting an odds ratio (OR) of 3.380, emerges as the most significant predictive factor for CRCI, aligning with insights from prior investigations^34^. Historical investigations have identified the principal causes of chemotherapy-induced cognitive impairment as encompassing: direct neurotoxic effects^35^ (Cytostatics penetrating the blood–brain barrier leading to cellular demise), hormonally induced alterations^36^ (Such alterations interfere with hormone secretion, precipitating cognitive difficulties, exemplified by chemotherapy-induced fluctuations in testosterone and estrogen levels, both considered neuroprotective hormones), oxidative stress^37^ (Chemotherapy reduces cellular antioxidant defenses, consequently amplifying DNA damage), cytokine-mediated immune dysregulation^38^ (Inflammatory cytokines traversing the blood–brain barrier may compromise cognitive faculties, evidenced by reduced processing speed, executive function, spatial ability, and reaction times), and vascular damage^39^ (Coagulation, vascular injury, and autoimmune events within the central nervous system’s microvasculature, with chemotherapy impeding cerebral microvascular blood flow). Despite the extensive body of research exploring the impact of chemotherapy on the incidence of CRCI in breast cancer, the precise mechanisms driving chemotherapy-induced cognitive impairments remain incompletely understood, warranting additional investigation. Furthermore, patients undergoing chemotherapy subsequently experience symptoms such as anxiety, depression, fatigue, and pain, which diminish their social engagement and physical activity, thereby detrimentally impacting their cognitive functions. Cognitive impairments resulting from chemotherapy typically arise from the complex interplay of various physiological and psychological mechanisms. Comprehending these mechanisms is pivotal for devising effective strategies aimed at preventing, identifying, and managing CRCI in patients with breast cancer. Healthcare professionals must closely monitor alterations in cognitive functions in patients undergoing chemotherapy and promptly implement appropriate interventions, such as narrative nursing and mindfulness-based stress reduction therapy.

#### Patient-centered experience: benefit finding and post-traumatic growth

This investigation delineates the discovery of benefits and post-traumatic growth as pivotal protective factors against Cognitive Impairment in Breast Cancer among patients. Within the domain of positive psychology, it has been observed that individuals can undergo significant positive psychological transformations, including the discovery of benefits and post-traumatic growth, following stressful or traumatic experiences, such as cancer. Studies by Chien WT and colleagues have substantiated that sustaining an optimistic disposition and a constructive mindset can beneficially modulate the body’s neuroendocrine system, mitigate the secretion of stress-induced hormones such as cortisol, thus augmenting immune system functionality, diminish anxiety and depression, alleviate fear, and ultimately facilitate the recovery of patients’ physical and psychological well-being^40^. Conversely, the identification of benefits and post-traumatic growth serves to bolster patients’ perceptions of self-efficacy. An amplified sense of self-efficacy can motivate patients to engage more proactively in health management and cognitive training endeavors and embrace beneficial lifestyle modifications such as consistent exercise, nutritious eating, and sufficient sleep, thereby effecting positive changes for the prevention or mitigation of cognitive impairment^41^. Consequently, healthcare practitioners and familial caregivers are urged to adeptly facilitate the genesis of positive psychological transformations in patients, enabling them to realize potential growth amidst adversity and effectuate beneficial alterations in their existence. However, to date, no empirical research has established a direct or indirect correlation between positive psychological determinants, such as the discovery of benefits and post-traumatic growth, and CRCI in breast cancer patients. In light of the extant research, this manuscript ventures to predict that post-traumatic growth and the identification of benefits might exert mediating or interactive influences on factors such as anxiety and fear, thus directly or indirectly impacting the incidence of CRCI in breast cancer patients.

#### Complex symptom: anxiety and fear of cancer progression

In alignment with our predictive model, prior studies have delineated anxiety and fear as significant risk factors for CRCI^42^. Research conducted by Bortolato B and colleagues^43^ has revealed that excessive anxiety and fear can compromise memory functions by concentrating attention on particular obsessions, with anxiety disorders potentially culminating in memory loss. Anxiety and fear can induce considerable psychological distress in patients, leading to an elevated release of cortisol from the adrenal glands, which adversely affects memory functions^44^. Notably, cortisol directly influences the hippocampus, an integral component of the limbic system responsible for working and short-term memory. The emotions of fear and anxiety compel patients to allocate significant attentional resources towards fear- and anxiety-centric thoughts, culminating in insomnia, fatigue, diminished appetite, and challenges in emotional regulation^44^. Moreover, individuals undergoing breast cancer treatment frequently exhibit anxiety and fear, attributable to various factors including surgical interventions, pharmacological treatments, and the intrinsic desire for life preservation^45^. Consequently, fear and anxiety, through their impact on patients’ physiological stress responses, attention distribution, sleep cycles, emotional regulation, neurobiological condition, and lifestyle choices, have been implicated in the development of CRCI. Consequently, comprehensive management of fear and anxiety, encompassing psychosocial support, the advocacy of a healthy lifestyle, and the implementation of professional psychological interventions, is crucial for the prevention and alleviation of CRCI. Future investigations are imperative to delineate the specific mechanisms of these therapeutic interactions, thereby enabling the formulation of more effective treatment strategies and ensuring more comprehensive care and support for breast cancer patients.

#### Biobehavioral factors: fasting blood glucose

Among biobehavioral determinants, fasting blood glucose levels have been delineated as a risk factor for Cognitive Impairment in Breast Cancer. Glucose functions as the principal energy substrate for cerebral cells, notably neurons, which exhibit the highest energy requisites^46^. Studies conducted by Luchsinger and colleagues^47^ have demonstrated that disorders in glucose metabolism can precipitate neurovascular unit dysfunction and dysregulation of blood flow, engendering oxidative stress in vascular endothelial and neuroglial cells, along with neuronal damage prompted by immune-inflammatory responses, consequently impinging upon patients' cognitive functions. Subsequent research has unveiled that diabetes and its ensuing complications, including hyperglycemia, hypoglycemia, and insulin metabolic dysregulation, exhibit a close association with CRCI. Nevertheless, the exact pathogenic mechanisms underpinning cognitive decline await comprehensive elucidation. Hyperglycemia may induce changes in neuronal osmotic pressure by modulating local cerebral blood flow, culminating in oxidative stress and ensuing neuronal damage^48^. Although the precise mechanisms underlying neuronal damage induced by hypoglycemia remain elusive, a population-based retrospective cohort study comprising 5966 patients with at least one documented episode of hypoglycemia has demonstrated an association with cognitive function impairment^49^. Insulin receptors are prolifically located within the hippocampus, olfactory cortex, and frontal lobes—areas integral to memory, attention, and executive functions^50^. This observation intimates that insulin may play a pivotal role in cognitive processes by modulating cortical activity and cerebral metabolism, potentially through the regulation of acetylcholine production, a key neurotransmitter. Furthermore, fluctuations in fasting blood glucose levels can serve as indirect indicators of treatment-related conditions. Abnormal fasting blood glucose levels may signify alterations in hormonal levels due to administered treatments, such as chemotherapy and endocrine therapy, thereby influencing blood sugar levels and, consequently, affecting cognitive functions directly or indirectly. Blood sugar levels in breast cancer patients frequently receive insufficient attention; thus, it is imperative for healthcare professionals to also focus on this aspect.

#### Phenotypic characteristics

In our study, the six predictors of Phenotypic characteristics—adherence, language function, hearing function, limb movement function, sleep function, and BMI—were all statistically significant in univariate analysis; however, they were not retained in the final predictive model, diverging from prior research findings^51–53^. One possible explanation could be the limited size of the sample; another is that symptoms in breast cancer patients during treatment typically do not manifest independently but are interrelated and synergistic, leading to the formation of symptom clusters. This synergy complicates the independent representation of related factors in phenotypic characterization. Consequently, future research should focus on the impact of symptom clusters on CRCI in breast cancer and broaden the study population to elucidate the relationships between relevant predictors of phenotypic representation and CRCI.

### Construction and application of a predictive model for cognitive impairment among Breast *cancer* patient

To our knowledge, this investigation constitutes the inaugural effort to formulate and appraise a user-friendly and comparatively personalized model for forecasting cognitive impairment in breast cancer patients. This research introduces a straightforward, personalized model for predicting CRCI, thereby facilitating the enhancement of clinical management practices. The model developed herein achieved an area under the curve (AUC) of 0.955 and a sensitivity of 0.906, denoting high discriminative capacity and accuracy. Protective factors against CRCI among breast cancer patients encompass residential locale, educational attainment, benefits realization, and post-traumatic growth, detailed as follows: educational attainment of 0–6 years (OR: 0.333) and beyond 6 years (OR: 0.103), benefits realization score (OR: 0.939), and post-traumatic growth (OR: 0.903). Risk factors entail chemotherapy (OR: 3.380), fear of disease progression score (OR: 1.042), anxiety score (OR: 1.298), and fasting blood glucose levels (OR: 1.660).

A recent study employed logistic regression models to develop a risk prediction model for chemotherapy-induced cognitive impairment among breast cancer patients^54^, derived from a multicenter cross-sectional analysis. This model was constructed using solely demographic and treatment-related data, comprising four variables in total. Our study systematically assesses the dynamic symptoms of CRCI and incorporates fasting glucose levels as a biobehavioral predictive factor. Our predictive model aims to enhance the accuracy of identifying high-risk breast cancer patients who have not yet manifested symptoms of CRCI. This nomogram can be integrated with existing relevant scales for clinical application. Individual breast cancer patients underwent comprehensive evaluation. For instance, a patient currently asymptomatic for CRCI might not reach the diagnostic threshold on relevant screening scales, thereby obscuring the probability of CRCI at this stage; our study addresses this gap. Our nomograms enable the estimation of an individual's risk of developing Cognitive impairment, facilitating a deeper understanding of the influence of various factors on CRCI, and aiding in the provision of personalized treatment and support. This, in turn, assists healthcare professionals in making informed decisions about beneficial interventions for breast cancer patients.

## Limitations

Our investigation is subject to several notable limitations. First, this was a cross-sectional study, which limited us in understanding the temporal sequence and determining the causality among variables. Furthermore, since all participants originated from the northern region of Anhui Province, this may constrain the generalizability of the sample. Secondly, our predictive model has not undergone external validation. Nonetheless, the internal validation via the Bootstrap resampling method and the calibration of predicted probabilities of CRCI in observed breast cancer patients constitute a strength of our study. Thirdly, owing to constraints inherent in the objective research conditions, certain biological and behavioral patient data, like intestinal flora, remain inaccessible. Lastly, the information related to the CRCI was all self-reported, this may lead to a bias of overestimation or underestimation of the actual measurement for CRCI. Future directions include undertaking multi-center, large-scale, longitudinal studies to enhance the validation and generalization of our findings.

## Conclusion

Our research is pioneering in applying the symptom scientific model to investigate CRCI in breast cancer patients, systematically and comprehensively examining the associated influencing factors. The findings indicate that residence area, educational level, chemotherapy, perceived benefits, post-traumatic growth, fear of cancer progression, anxiety, and fasting blood glucose independently contribute to CRCI. Previous research has predominantly focused on the negative psychosocial factors and treatment methods associated with CRCI, which is a dynamically systematic process. However, these studies have not adequately addressed the comprehensive evaluation and construction of predictive models for CRCI. Our study addresses this gap by providing a comprehensive assessment and predictive modeling of CRCI in breast cancer patients. Building on previous research, our study not only explores the correlation between benefit findings, and positive psychological changes linked to post-traumatic growth, and CRCI but also identifies the objective predictive role of biobehavioral factors such as fasting blood glucose in breast cancer CRCI. Concurrently, we constructed a nomogram model for breast cancer cognitive impairment, characterized by high discrimination and accuracy.

As data science and artificial intelligence technology continue to advance, clinical, imaging, biobehavioral, and patient self-reported data will see continuous improvement. The integration of multi-modal data and big data analysis will become pivotal in studying CRCI, allowing for a deeper exploration of the factors influencing CRCI in breast cancer patients. Predictive models in this field will undergo continuous optimization. It is anticipated that individualized interventions and support programs will be more effectively implemented, significantly enhancing the quality of life for breast cancer patients.

## Data Availability

The datasets generated and/or analyzed during the current study are not publicly available because of the privacy implications but are available from the corresponding authors on reasonable request.
